# Cascaded plasmon-plasmon coupling mediated energy transfer across stratified metal-dielectric nanostructures

**DOI:** 10.1038/srep34086

**Published:** 2016-10-04

**Authors:** Sepideh Golmakaniyoon, Pedro Ludwig Hernandez-Martinez, Hilmi Volkan Demir, Xiao Wei Sun

**Affiliations:** 1 LUMINOUS! Center of Excellence for Semiconductor Lighting and Displays, School of Electrical and Electronic Engineering, Nanyang Technological University, 639798 Singapore; 2 Division of Physics and Applied Physics, School of Physical and Mathematical Sciences, Nanyang Technological University, 21 Nanyang Link, 637371 Singapore; 3 Department of Electrical and Electronic Engineering, College of Engineering, South University of Science and Technology, 1088 Xue-Yuan Road, Shenzhen, Guangdong, 518055 China

## Abstract

Surface plasmon (SP) coupling has been successfully applied to nonradiative energy transfer via exciton-plasmon-exciton coupling in conventionally sandwiched donor-metal film-acceptor configurations. However, these structures lack the desired efficiency and suffer poor photoemission due to the high energy loss. Here, we show that the cascaded exciton-plasmon-plasmon-exciton coupling in stratified architecture enables an efficient energy transfer mechanism. The overlaps of the surface plasmon modes at the metal-dielectric and dielectric-metal interfaces allow for strong cross-coupling in comparison with the single metal film configuration. The proposed architecture has been demonstrated through the analytical modeling and numerical simulation of an oscillating dipole near the stratified nanostructure of metal-dielectric-metal-acceptor. Consistent with theoretical and numerical results, experimental measurements confirm at least 50% plasmon resonance energy transfer enhancement in the donor-metal-dielectric-metal-acceptor compared to the donor-metal-acceptor structure. Cascaded plasmon-plasmon coupling enables record high efficiency for exciton transfer through metallic structures.

Energy transfer (ET) from an excited donor dipole to an acceptor may occur either radiatively or nonradiatively. Radiative transfer corresponds to emission and absorption of photons, in which the average distance between the donor and the acceptor should be larger than the donor photoemission wavelength, while nonradiative energy transfer (NRET) needs a distance much shorter than the photoemission wavelength (for dipole-dipole interaction in the range of 1–10 nm) and arises without emission of “real” photons^1^,^2^,^3^,^4^,^5^,^6^,^7^,^8^. However, utilizing the NRET mechanism within the moderate separation distance (>10 nm) between the donors and the acceptors lacks the desired efficiency. Instead, surface plasmon mediated nonradiative energy transfer (SP-NRET) has been demonstrated to increase the NRET efficiency beyond the Förster radius limit.

SP-NRET results from the strong near-field coupling of dipole moment with the surface plasmon modes of the metallic-dielectric interface, which considerably modifies the energy transfer efficiency. SP-NRET offers great potential in numerous applications such as biosensing, light harvesting and light generation^9^,^10^,^11^,^12^,^13^. In particular, emission enhancement of molecular fluorescence near metallic nanostructures and increased energy transfer through the plasmonic nanostructures have been widely exploited^5^,^6^,^7^,^13^. Furthermore, localized surface plasmon mediated NRET through metal nanoparticles has been demonstrated to effectively increase the Förster radius; and emission enhancement has been obtained in a precisely-designed emitting structure^7^. Since the very first study on SP-NRET through optically thick/lossy metal films^14^, several applicable systems have been verified to enhance the efficiency of the process and extend the effective ET range^2^,^5^,^15^,^16^. Despite the fact that SP improves the ET range up to 150 nm through a silver film^17^, the mechanism suffers from the significant energy loss due to the absorption by thick metal film.

In this study, we propose and demonstrate the nonradiative energy transfer mechanism between the donor and the acceptor multilayers through layered metallic nanostructures, in which the stratified configuration gives rise to an efficient energy transfer process. This novel approach in NRET, uniquely provides us with the ability to overcome the drawback of high energy absorption losses in a thick metal film by inserting a non-absorbing dielectric layer between two thin metal films. Moreover, a strong plasmon-plasmon near-field coupling through the dielectric spacer layer is proven to highly extend the effective ET distance/efficiency. Alternatively, placing dielectric and metal layers instead of a single thick metal film allows for the SP modes to overlap efficiently through layered configurations. The proposed architecture leads to a remarkable energy transfer efficiency as a result of stronger surface plasmon coupling at the metal-dielectric boundaries.

## Results

### SP-NRET in plasmonic nanostructures

The mechanism of SP-NRET goes beyond the conventional Förster resonant energy transfer process since the direct interaction of the donor and the acceptor dipoles moments is not effective in SP-NRET. As a matter of fact, the evanescent electric fields coupling of the donor/acceptor excitons to the surface plasmon modes of the metal films plays a crucial role in energy migration^3^,^18^,^19^,^20^. It was well demonstrated in literature that the key factor in designing the structures benefiting an efficient SP-NRET, is to distinguish the difference between the direct quenching/enhancing effect of a plasmonic nanostructure on the donor/acceptor emission and the improved efficiency due to SP-NRET^5^,^9^,^21^,^22^. Therefore, a perceptive understanding of the overall processes is essential to realize an efficient SP-NRET in nanostructures.

**Figure 1:**
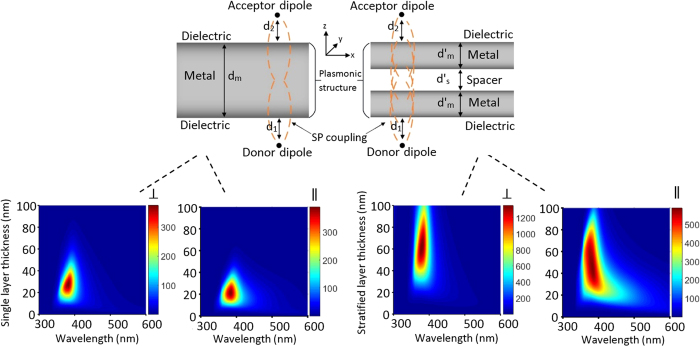
Single layer and stratified nanostructures and enhancement factor. Architectures of the single layer (**a**) and stratified (**b**) plasmonic nanostructures. The donor dipole is placed at one side of the plasmonic layer (single metal film or stratified metal-dielectric layers) and the acceptor dipole is at the opposite side. The surrounding media are isotropic and non-absorbing. Calculated electric field enhancement factor at the acceptor dipole position as the functions of plasmonic layer thickness and excitation wavelength for simple (**c**,**d**) and stratified (**e**,**f**) nanostructures and theoretical steady state efficiency. The dipole moment is oriented perpendicular (**c**,**e**) and parallel (**d**,**f**) with respect to the plasmonic layer surface. The donor/acceptor dipole distance to the nearest plasmonic layer surface is 10 nm and the equation 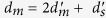 is applied for the single layer and stratified configurations.

This study aims to apply a stratified structure of metal-dielectric-metal instead of a single metal film to benefit from not only stronger stratified evanescent SP coupling but also the reduction of energy absorption losses. In this article, the stratified SP coupling refers to the cross-coupling of an excited SP modes at each of the metal-dielectric interfaces. This electromagnetic coupling arises from an interaction of an excited oscillating dipole moment with the surface modes of metal film. Since the evanescent electric field emission of a dipole acts as an excitation source to derive the surface plasmon modes at the interfaces, the theoretical simulation of electric field enhancement across all layers, presents the capability of the structure to enhance/suppress the SP couplings^15^,^20^,^21^,^23^. The geometry for the single metal film and the stratified structures is depicted in Fig. 1a,b, respectively. The donor and the acceptor dipoles are placed on either side of the metal film in the single layer structure and the stratified layer configuration (metal-dielectric-metal).

The first step of SP-NRET process can be explained through the Sommerfeld’s theory of an excited oscillating dipole near an absorbing medium^24^. The excitation of a donor dipole leads to the near-field electromagnetic emission^25^,^26^,^27^ of a dipole moment in which the short range dipole electric field is coupled to the surface modes of the metallic film and the energy of an excited donor dipole is nonradiatively transferred to surface plasmon modes of the metal film^3^,^28^,^29^. The second step of SP-NRET needs to be describe separately for the single layer and stratified configurations. In the case of single layer structure, the SP modes at the interfaces of each side of the metal film are cross-coupled and the energy is transported to an acceptor dipole. On the other hand, in stratified structure, the cross-coupling takes place across the SP modes of metal-dielectric and dielectric metal interfaces. We should point out that, while the evanescent interaction of an oscillating dipole with SP modes of the metallic film comprises all the Fourier components, the dipole near-field should be treated differently in comparison with the radiation field (see Methods). Moreover, the exponential dependency of the energy flux on the skin depth in the case of radiation field cannot be applied for the near-field coupling^29^. As a matter of fact, the enhanced electric field at the metal-dielectric interfaces reflects the ability of the plasmonic nanostructure in mediating energy transfer from the donor to the acceptor dipoles (Supplementary Fig. 1).

The plasmon-mediated nonradiative ET rate is obtained by the classical electrodynamics model^23^ expressing the enhancement factor as the ratio of an induced electric field at the acceptor dipole position in the presence and absence of a plasmonic nanostructure:


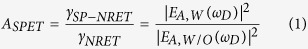


where *A*_*SPET*_ is the plasmonic enhancement factor; *E*_*A,W*_ and *E*_*A,W*/*O*_ are the induced electric field at the acceptor position with and without the plasmonic nanostructure, respectively; 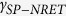 is the nonradiative ET rate to the acceptor in the presence of the plasmonic structure; and *γ*_*NRET*_ is the nonradiative ET rate from the donor to the acceptor. Figure 1 depicts the calculated enhancement factor versus single metal film/stratified metal-dielectric thickness and wavelength for the perpendicular (Fig. 1c,e) and the parallel (Fig. 1d,f) dipoles (color map plots). The thickness of single metal film (*d*_*m*_) in the single layer structure and the metal-dielectric-metal layer thickness (2*d*′_*m*_ + *d*′_*s*_) in stratified configuration, varies from 0 to 100 nm. The distance of the donor and the acceptor dipoles to the nearest metal surface; *d*_1_ and *d*_2_ (Fig. 1a,b) are 10 nm. In this simulation the thickness of two metal films (*d*′_*m*_) and the spacer layer (*d*′_*s*_) are equally distributed in the stratified configuration, since it has been demonstrated by numerical simulations that this choice results in the maximum enhancement factor (Supplementary Fig. 2). The permittivity of the silver metal films was taken from Johnson and Christy^30^ dielectric function. In order to fulfil the resonance matching condition in all the metal–dielectric interfaces, the similar refractive index of n = 1.7 was chosen for dielectric layers (the donor, the acceptor and the spacer layers). Noted that the resonance matching condition arises from the most efficient cross-coupling of SP modes at each interfaces that leads to the highest enhancement factor and can be expressed as a function of the metal and the dielectric layers refractive indexes^3^,^9^,^20^,^29^.

As can be seen in Fig. 1, the resonance matching condition for perpendicular/parallel dipole orientations and stratified/single layer structures occurs at the wavelength of 380 nm at which the real part of the silver dielectric function equals to the spacer dielectric constant in opposite sign. The maximum enhancement factor in the single layer structure occurs at the silver film thickness of 28 and 22 nm for perpendicular and parallel dipoles, respectively, indicating the optimum silver thickness for the strongest SP cross-coupling (Fig. 1c,d). On the other hand, the stratified layer thickness of 61 and 50 nm (including the two metal films and the dielectric film) corresponds to the highest enhancement factor in the stratified configuration for perpendicular and parallel dipoles (Fig. 1e,f). To the best of our knowledge, the physical origins of these optimum thicknesses can be explained through the fact that a very thick metal film suffers from weak overlaps of SP modes at the interfaces due to the long distance between them, while in a very thin metal film the SP modes are not strong enough to efficiently enhance the induced electric fields at the acceptor position through the interfaces. This claim can be verified through an electric field distribution calculation across the layers in which the highest enhancement factor at the metal-dielectric interfaces has been obtained in the structure with an optimized metal film thickness that leads to the maximum enhancement factor at the acceptor position (Supplementary Fig. 3). The numerical calculation suggests that if the metal film is too thin, the electric field at the metal-dielectric interfaces cannot be sufficiently enhanced and in the structure with a very thick metal film the interfaces are too far from each other to be able to considerably increase the electric field at the acceptor dipole position.

Regardless of the dipole orientation, the maximum enhancement factor is increased up to 3 fold in the stratified configuration compared to the single metal layer structure. Thus, the stratified structure induces higher electric field at the acceptor dipole giving rise to higher SP-NRET efficiency. It is worth mentioning that different choices of *d*_1_ and *d*_2_ (Fig. 1a,b) lead to the same conclusion that plasmonic effect is enhanced in the stratified configuration (Supplementary Fig. 4). In addition to the fact that the enhancement factor simulation predicts the higher SP coupling in the plasmon-plasmon stratified nanostructure, the nonradiative and radiative decay rates of the donor/acceptor dipoles were taken into account for modelling the overall SP-NRET mechanism in our simulation (Supplementary Fig. 5). More details on decay channels and efficiency calculation is covered in the Methods section.

### Device geometry

The single metal layer and the stratified samples consisting of the donor, the plasmonic nanostructure, and the acceptor layers are fabricated on quartz slides. The thickness of the donor and the acceptor layers are 15 nm for all the sample geometries. The plasmonic nanostructure consists of either a single evaporated silver thin film or stratified layers of silver films and a dielectric layer. The silver films are deposited using thermal evaporation system with a minimum thickness of 20 nm to form homogeneous layers rather than formation of nano-islands^29^. A quenching control layer was deposited between the donor/acceptor and the nearest metal film to maximize the ET rate as the interaction of the oscillating dipole electric field with the SP modes of the metal, highly depends on the dipole distance to the metal surface^2^,^6^,^31^.

**Figure 2:**
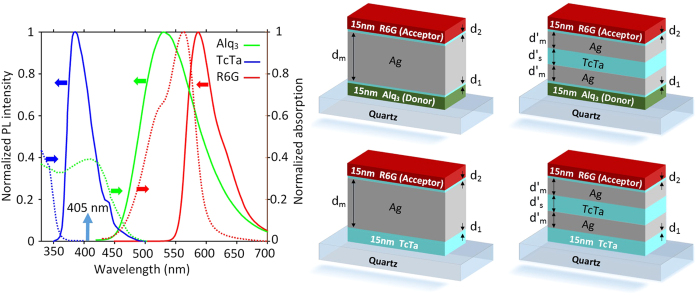
Emission/Absorption spectrum. (**a**) Absorption (dashed line) and photoluminescence (solid line) spectra for the donor: Alq3 (green), the spacer: TcTa (blue), and the acceptor: R6G (red). The vertical arrow indicates excitation laser wavelength for PL measurements. (**b**,**c**) single layer and stratified structures, and their respective control samples (**d**,**e**).

The key factor in an efficient nonradiative energy transfer is that the emission spectra of the donor should overlap the absorption spectra of the acceptor, hence several combinations of dyes have been studied in literature^14^,^17^,^32^,^33^; the pair of Alq3, Tris (8-hydroxyquinolinato) aluminum and R6G, Rhodamine 6G is one the most efficient pair for our purpose. The absorption and emission spectra for the donor, the acceptor, and the spacer layers are depicted in Fig. 2a. Alq3 was used as the donor with the emission peak at 525 nm which overlaps with the absorption peak of R6G as the acceptor. TcTa, Tris (4-carbazoyl-9-ylphenyl) amine, was used as the spacer layer between the two silver films and the quenching control layer between the donor/acceptor and the silver film. Consequently, the structures for the stratified (Fig. 2c) and the single metal layer (Fig. 2b) samples are glass/Alq3/TcTa/Ag/TcTa/Ag/TcTa/R6G and glass/Alq3 /TcTa/Ag/TcTa/R6G, respectively, and their respective control samples (Fig. 2d,e) have been obtained by replacing the donor layer (Alq3) with spacer layer (TcTa).

### Photoluminescence and time resolved characterizations

**Figure 3:**
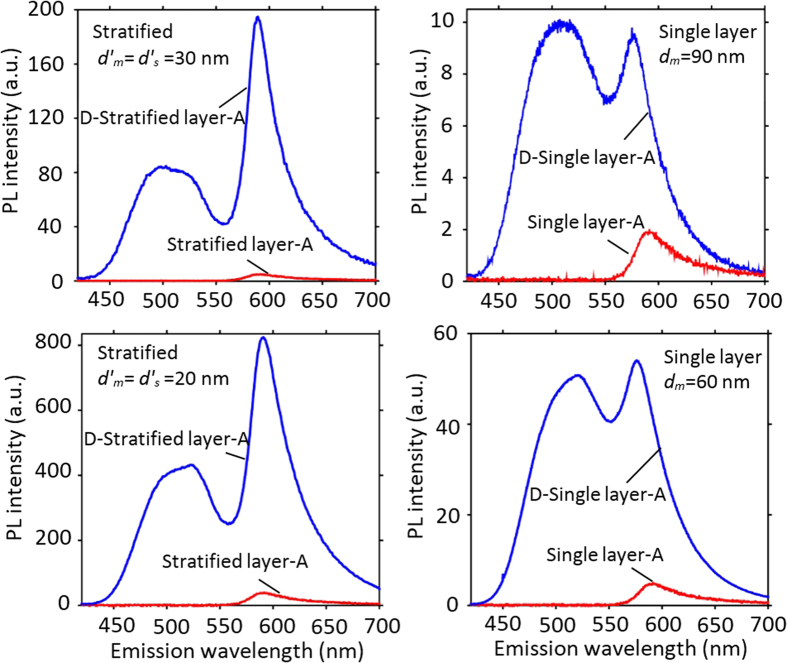
PL intensity. PL intensity from the stratified (**a**,**c**) and single layer (**b**,**d**) structures for single/stratified layer-acceptor (red) and donor-single/stratified layer-acceptor (blue) samples. In the single layer structure the thickness of silver film and in the stratified samples the metal-spacer-metal thickness is 60 (**c**,**d**) and 90 nm (**a**,**b**). The excitation source is 405 nm. The stratified samples show the higher SP-NRET efficiency and absolute photoemission compared to the single layer structures.

Room-temperature steady-state photoluminescence (PL) spectra for the single layer, the stratified and the control samples with the plasmonic layer thickness of 60 and 90 nm (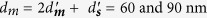), are shown in Fig. 3. Noted that for all the samples, a 405 nm excitation laser source was applied from the donor site (spacer site in control samples) and the PL intensities were collected from the acceptor side. From the control samples PL spectra (red line), it can be verified that the direct excitation of the acceptor with the laser source has a negligible effect on the acceptor excitation since the acceptor PL intensities with the peak emission wavelength of 585 nm are distinctly small compared to acceptor emission in corresponding single layer and stratified samples (blue line). As a matter of fact, the significant presence of the acceptor emission in the donor-single layer-acceptor and the donor-stratified layers-acceptor samples is the direct effect of SP-NRET from the donor to the acceptor site, demonstrating the role of the plasmonic structure as the energy mediator. In other words, from the two sources of excitation that are available for the acceptor layer; the external laser source and the SP-NRET from donor layer, the latter one has the major contribution in the acceptor excitation. Furthermore, it can be clearly seen from Fig. 3 that the acceptor emission is significantly increased in the stratified structures compared to the corresponding single layer samples. The ratio of the acceptor emission in the presence of the donor layer (*I*_*AD*_) to the acceptor emission in the absence of the donor (*I*_*A*_) for the single layer structures with *d*_*m*_ = 60 and 90 nm is 11.25 and 4.94 (Fig. 3b,d), respectively; while this ratio for the stratified samples with *d*′_*m*_ = 20 and 30 nm is increased to 38.4 and 44.4 (Fig. 3a,c), respectively. It is worth noticing that the higher PL intensity in the stratified structures distinctly confirms the reduced absorption as a result of introducing the spacer layer between the metal films.

In order to measure the SP-NRET efficiency for the single layer and the stratified nanostructures, since the ET process takes place at two steps, both the donor and the acceptor photoluminescence intensities should be reflected in energy transfer efficiency calculation to fully cover the effects of each step on the donor and the acceptor PL intensities. Notably, the method described in Equation (2) provides an accurate SP-NRET efficiency measurement as a result of its dependency on the donor emission in the presence of the acceptor (quenched donor PL intensity) and the acceptor emission in the presence of the donor (sensitized acceptor PL intensity)^4^. It is worth mentioning that the conventional methods for measuring NRET efficiency may not be essentially effective for multi-step ET process as they involve an extra correction factor definition due to the fact that these methods are mainly based on the ratio of the donor PL intensity quenching in the presence and the absence of the acceptor^14^,^17^. Therefore, taking into account both the donor and the acceptor PL intensities, the ET efficiency can be expressed through the quenched donor and the sensitized acceptor intensities method as


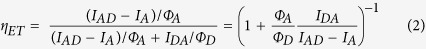


where *I*_*AD*_ and *I*_*A*_ are the acceptor PL intensities in the presence and the absence of donor, respectively; *I*_*DA*_ is the donor PL intensity in the presence of acceptor; and 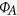 and 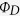 are the acceptor and the donor intrinsic quantum yields, respectively. Since the ET mechanism is a unidirectional process from the donor to the acceptor through the plasmonic nanostructure, the excitation of the acceptor and accordingly its energy relaxation could not lead to the donor excitation. Therefore, the donor PL intensity is the result of an external excitation that is modified through its vicinity to the plasmonic nanostructure, while the acceptor emission is obtained according to the sum of an external excitation (laser source) and the SP-NRET. As a result, in order to study the effect of SP-NRET on the acceptor emission, the acceptor photoluminescence cause by the laser excitation source should be extracted from the overall acceptor emission (*I*_*AD*_ − *I*_*A*_). After the spectral decomposition of the donor and the acceptor photoemission from the mixed emission spectra, the terms *I*_*DA*_ and *I*_*AD*_ can be defined as the area under the donor and the acceptor spectra, respectively.

**Figure 4:**
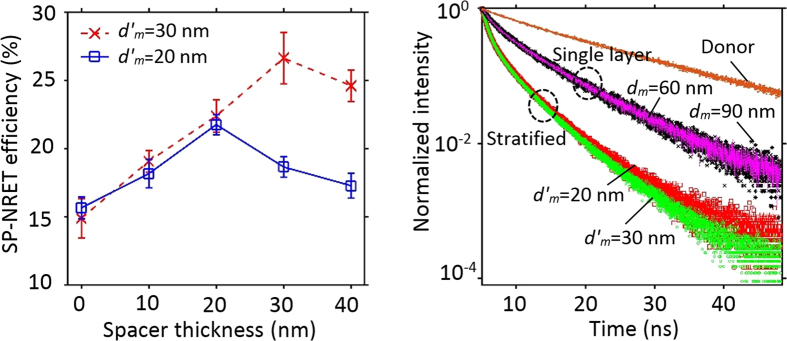
Spacer optimization and time resolved spectroscopy. (**a**) SP-NRET efficiency in the stratified nanostructures with *d′*_*m*_ = 20 and 30 nm versus the spacer layer thickness. The highest SP-NRET efficiency is obtained at the thickness of the spacer layer equals to the silver films for each set. (**b**) Time resolved photoluminescence decay curves with two-exponential decay model at the donor emission wavelength for the donor-only layer (Alq3), the single layer structures with *d*_*m*_ = 60 and 90 nm and the stratified configurations with 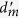 = 20 and 30 nm. Faster decay rates is observed in the stratified samples compared to the single layer structures. In the stratified structures, the thickness of silver layers and the spacer layer are deposited equally (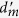 = 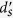).

For the purpose of the comparison of SP-NRET efficiency in the single layer and stratified configurations, we consider two cases: (1) the thickness of plasmonic nanostructure in the stratified configuration is equal to the silver film in single layer structure (*d*_*m*_ = 2*d*′_*m*_ + *d*′_*s*_), which we used above; and (2) the total thickness of silver films in the stratified samples is equal to the thickness of silver film in the single layer structure which stands for the same energy absorption losses in plasmonic layer (*d*_*m*_ = 2*d*′_*m*_). Table 1 shows that regardless of the comparison method, the SP-NRET efficiency in the stratified samples is clearly higher than that in the single layer structures. For instance, in the stratified sample with 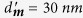 the average experimental efficiency is obtained 26.46%, which is 1.78 and 3.11 times greater than the single layer structures with 60 and 90 nm silver film thickness, respectively. In the case of stratified structure, the optimized thickness of the spacer layer was determined to obtain the highest efficiency. For instance in the stratified structure with 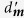 = 20 nm, by means of varying the spacer layer thickness from 0–40 nm, the maximum SP-NRET efficiency happens at 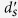 = 20 nm (Supplementary Fig. 6). As can be seen in Fig. 4a, the maximum SP-NRET efficiency occurred at the spacer thickness of 20 and 30 nm for stratified nanostructures with each silver film thickness of 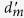 = 20 and 30 nm, respectively, which agrees well with the theoretical simulation (Supplementary Fig. 2).

Regardless of the fact that the photoluminescence characterization of the samples verify the presence of SP-NRET in our structures, time-resolved spectroscopy method has been also known as an unequivocal demonstration of ET mechanism. In simple donor-acceptor structures, if the lifetime of the donor is significantly longer than that of the acceptor, the donor lifetime decreases in the presence of nonradiative energy transfer from the donor to the acceptor. As a matter of fact, ET process gives rise to an additional nonradiative decay channel for the donor molecule that raises the overall decay rates and consequently reduces the donor lifetime. However, in the plasmonic nanostructures the modification of the donor lifetime should be interpreted differently as the presence of the acceptor molecule may not considerably modify the donor lifetime^14^,^17^. More specifically, in our structure the distance between the donor and the acceptor layers is large enough to prevent any direct nonradiative energy transfer from the donor dipole to the acceptor dipole and the modification of the donor decay rate occurs due to the ET from the excited donor molecule to the SP modes at the metal surface (the first step of SP-NRET).

Temporal evolution of spectrally resolved donor emission was collected for all the samples using an appropriate bandpass filter to bypass the acceptor emission. The time resolved PL for the donor-only, single layer and stratified nanostructures at the donor emission peak wavelength are shown in Fig. 4b. Donor-only sample exhibits a decay lifetime of 13.5 ns that is in good agreement with previous studies^34^,^35^. In the case of single metal film layer and stratified films, since the relaxation of the excited donor molecules occurs in front of different local photonic environments for each of the molecules, a more complex temporal behavior has been expected from the decay curves. Two exponential fitting was employed for amplitude average lifetime approximation of the single layer and the stratified nanostructures. The amplitude averaged decay lifetime of the donor in single metal film layer sample with silver film thickness of 60 and 90 nm were obtained 1.95 and 1.96 ns, respectively. In stratified structures the measured lifetime decreases down to 0.85 and 0.89 ns for 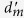 = 20 and 30 nm, respectively. Notably, the significant reductions of donor lifetime in the single layer and stratified structures confirm the presence of very large nonradiative decay channels as a result of SP modes on metal surface. Moreover, shorter donor lifetime in stratified structures compares to single layer sample demonstrates faster nonradiative ET rate from donor layer to the SP modes, which arises from the capability of the stratified layers in enhancing the SP modes coupling and consequently increasing the decay rates of the donor. As mentioned before, our experimental observations show that the presence/absence of the acceptor layer has negligible contribution in the donor temporal behavior (Supplementary Fig. 7). As a result, it is important to emphasize that the temporal behavior of the donor emission is only the evidence of ET to SP modes while for the comprehensive study of SP-NRET mechanism, the effect of SP cross-coupling (which is expressed by enhancement factor) should be also taken in to account. More detailed results on time-resolved measurements and simulations for single layer and stratified structures are presented in Table 2. Noted that the theoretical calculation were performed base on the modification of both radiative and nonradiative decay channels to estimate all the decay rates (see Methods).

## Discussion

Through numerical simulation of the donor dipole induced electric field at the acceptor position, we show that the stratified plasmonic nanostructure leads to a higher enhancement factor compared to the single layer configuration. Since the SP-NRET mechanism is highly dependent on the plasmon-induced electric field at the metal-dielectric interfaces^3^, introducing an extra interface in the stratified plasmonic nanostructure amplifies the structure capability to enhance the electric field. We consider this factor as the ability of plasmonic nanostructure in transferring the nonradiative energy from the donor to the acceptor. In order to simulate the overall process of SP-NRET from the excited donor dipole to the acceptor dipole through the plasmonic nanostructure, we developed a model based on CPS-Kuhn theory, which expresses the decay rate constant of an excited dipole through the radiative and nonradiative components due to the effect of the absorbing medium^3^ (see Methods). The context of this model was derived from the classical electrodynamics theory of a nano-antenna near earth that was modified to define the decay rates of an oscillating dipole in adjacent of the metallic nanostructure^3^,^24^,^28^,^29^,^36^,^37^. However it has been shown through considering accurate overlap function of a guest emission and a host absorption, that the classical and quantum approaches can be treated equivalently^28^.

The SP modes at the metallic nanostructure is first considered as the primary acceptor site for the donor dipole to calculate the nonradiative ET transfer rate. Next, the induced electric field of the donor dipole at the acceptor dipole position is taken as the enhancement factor for ET rate to excite the secondary acceptor. Similar numerical calculation was applied to the excited acceptor dipole to separately derive the radiative and nonradiative decay rates. Note that, to evaluate the theoretical results for experimental verification, further modification is needed as the theory analysis is performed for single donor dipole energy transfer to the single acceptor dipole through the plasmonic layer, while in the experiment, thin film form of donor and acceptor was used. To model and quantify the multilayers of the donor/acceptor in the simulation, the 15 nm donor/acceptor thin films was taken as 15 layers of donor/acceptor dipoles stacked on each other (the dye dipole diameter ~1 nm). Consequently, the two-dimensional dipole arrayed structure was applied at each step of ET calculation^15^. Another important assumption is that the homo transfer between the donor/acceptor dipoles was neglected and only the hetero transfer of the donor and the acceptor was considered. The simulation results showing in Table 1 are obtained for the isotropic distribution of the donor/acceptor dipole. As can be seen from this table, the theoretical and experimental results are in good agreement confirming higher SP-NRET efficiency enhancement in the stratified nanostructures compared to the single layer samples. The slight inconsistency in SP-NRET efficiency from experiments and theory can be explained through the following assumptions that we have considered in our theoretical calculations: first, the smooth surface approximation in the model, while in reality the surface roughness of silver as a result of thermal deposition is inevitable which may introduce high scattering; second, the two-dimensional assembly model for the donor and the acceptor layers can’t be precisely realized in experimental samples. We assumed in our calculation that dipoles arrays are uniformly stacked on each other however, the random distribution of the donor/acceptor dipoles is more prone to take place.

In summary, we propose the stratified plasmonic coupling to enhance the SP-NRET in metal-dielectric nanostructures. Both experimentally and theoretically, we demonstrated that the stratified plasmonic layer leads to the higher ET efficiency compared to the single metal film nanostructure. Our findings show that multi-layer plasmonic configurations enhance the induced electric field of the emitting donor molecule because of surface plasmon near-field coupling created at the interfaces.

## Methods

### ET efficiency calculation

We derived convenient equations to calculate ET efficiency for the simple and the stratified plasmonic nanostructures. Due to the multi-step ET mechanism, we first build up our model to simulate the effect of a plasmonic nanostructure on the decay rates of the emitting donor dipole. It has been studied theoretically and experimentally that the metallic nanostructure noticeably modifies the nonradiative decay rate of an adjacent dipole via introducing the plasmonic coupling modes of a metal surface with the evanescent field of a dipole^7^,^20^,^21^. As a result, the nonradiative energy transfer to the SP modes of the metal becomes a main decay channel and gives rise to the large reduction in dipole lifetime. In order to evaluate the performance of the structures, we employed the theoretical model based on CPS theory of an excited dipole near a metal surface. Briefly, the electric and magnetic fields of an excited dipole is expressed by defining the Hertz vectors at the region of interest as 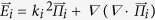 and 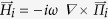. where 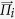 is the Hertz vector and *k*_*i*_ is the propagation constant at region *i*. Consequently, the boundary conditions at each interface is applied to simulate the near field surface modes between the metal and the dielectric layers. The Poynting vectors can be formulated using the electric and magnetic fields 
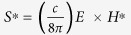
 which allows defining the total energy flux through all the layers by 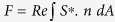. The decay constant is obtained as the ratio of the total energy flux to the total energy of the dipole. The distinct and notable advantage of this approach is that explicitly shows the radiative and nonradiative decay rates of an emitting dipole, based on the separation of the trivial and evanescent energy transfer terms in the decay rate expression. The approach is unique for each dipole, parallel (||) or perpendicular (⊥) to the interfaces, and can be extended to an isotropic (*iso*) one by applying 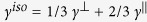, in which *γ* is the decay rate for different dipole orientations. Following the CPS model, we initially define the Hertz vectors in cylindrical coordinates (*r*, *z*, *φ*) for parallel and perpendicular dipoles respectively as


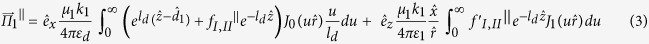



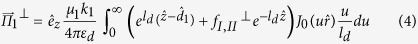


where *μ*_1_ and *ε*_*d*_ are the donor dipole moment and dielectric constant respectively; *k*_1_ is the wavenumber at the dielectric medium; 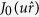 is the zeroth-order Bessel function; 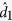 is the donor dipole distance to the surface of the plasmonic structure which is normalized to *k*_1_; 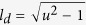; *u* is the integration variable and corresponds to normalized wavevector; the indices *I* and *II* refer to simple and stratified plasmonic nanostructure respectively, and 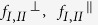
and 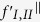 are the reflection factors calculated by applying the appropriate boundary conditions at the metal/dielectric interfaces. At this point, we are able to define the decay rates by employing the energy flux method and calculate the total energy flowing away from the donor dipole. Noted that for the sake of simplicity, the decay rates were normalized to the free space dipole decay rate. Therefore, based on the far field and near field distance dependency, the normalized radiative and nonradiative terms can be assigned. The results for the perpendicular donor dipole at the donor peak emission wavelength (*ω*_*D*_) for the two configurations are


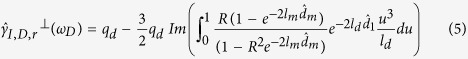



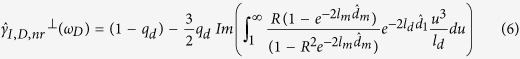



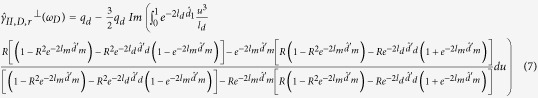



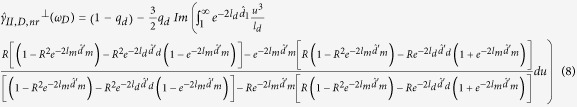


where *q*_*d*_ is an intrinsic quantum yield of the donor in the absence of the plasmonic structure, 
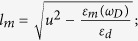
*R* is the p-polarized reflection coefficient equals to 
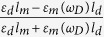
. Note that the first and the second terms (the integral expression), in Equations (5, 6, 7, 8), refers to an intrinsic decay rates of the dipole in the absence of the metallic mirror and the normalized decay rates modification due to the presence of the metallic nanostructures, respectively. Consequently, the total normalized decay rate of an emitting donor dipole can be expressed as 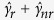. We should stress that the key factor in separating the total decay rate constant to radiative and nonradiative components, is the variation of the integral limits. As one can see in Equations (5) and (7), in which the normalized radiative decay rates of the dipole have been expressed for simple 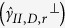 and stratified 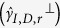 nanostructures, the integral limits varies from 0 to 1, while for the normalized nonradiative decay rate expression in Equations (6) and (8), the integral limits varies from 1 to ∞. This can be explained through the dipole behavior near the metal surface, which includes both, the far (0 < *u* < 1) and evanescent fields (*u* > 1), the corresponding region can be independently defined versus *u* to cover either the real (0 < *u* < 1) or complex (*u* > 1) angles of incidence^3^,^17^.

Next Equation (1) is applied to define the SP-NRET rate equation which refers to the second step of energy transfer from the SP modes of the plasmonic nanostructure to the acceptor. As discussed in the main text, the following assumption was taken as a result of the far distance between the donor and the acceptor molecules: at the first step of ET from the donor to the SP modes of metal, the possibility of direct nonradiative energy transfer from the donor to the acceptor is negligible. In other words, the plasmonic medium is responsible for mediating ET between the donor and the acceptor. The overall decay rates for each step are given by:


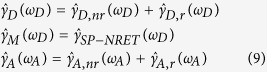


where 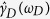 corresponds to the total donor dipole decay rate modification at the donor peak emission wavelength (*ω*_*D*_) which is equal to the sum of the radiative (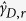) and the nonraditive (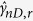) decay rates; 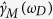 corresponds to the ET rate from the donor dipole to the SP modes of the plasmonic structure that is defined through electric field enhancement factor, i.e., 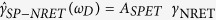; and 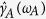 is the total acceptor dipole decay rate modification at the its peak emission wavelength (*ω*_*A*_). Following the steady-state condition, we define the peak intensity of the donor and the acceptor based on the decay rates equations. The ET mechanism is a unidirectional process from the donor to the acceptor through plasmonic layer, therefore the donor PL intensity is the result of an external excitation that is modified through its vicinity to the plasmonic structure, while the acceptor emission is obtained according to sum of external excitation and ET.


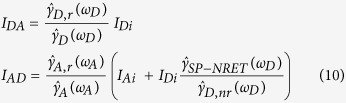


### Sample fabrication

The quartz substrates were cleaned using an ultrasonic bath with detergent, deionized water, acetone and isopropyl alcohol sequentially for 20 min and then dried by Nitrogen gas. The silver films and organic nanolayers were deposited using thermal evaporation system under the pressure of 4 × 10^−4^ Pa. The silver deposition rate was controlled (0.1 Å/s) to gain smooth surface and prevent an unwanted roughness. Similarly, all the organic layers, Alq3, TcTa, and R6G were thermally evaporated with a rate of 0.5 Å/s. The substrates were quartz slides.

### Photoluminescence and time resolved measurement

The linear steady state photoluminescence spectra of the samples were recorded using Shamrock SR-303i spectrograph that is integrated with Andor’s CCD camera spectroscopy detector systems. The excitation source was a diode laser with a wavelength of 405 nm. The excitation was applied from the donor side and the PL intensities were collected from the acceptor side in all the samples. The fluorescence lifetime measurements were carried out using a time correlated single photon counting system with an excitation laser at 375 nm. The band pass filter with a full width at half-maximum of 50 nm and central wavelength of 520 nm is used to record the decay rates of the samples at the donor emission. The time-resolved emission data were fitted with two-exponential approximation by Fluo-fit decay analysis software (Pico Quant Technologies, Germany). In the time-resolved system the pulse duration is 40 to 90 ps, repetition rate is 20 MHz, the CW- equivalent power is 0.3–2.5 mW and the collection N.A is 0.75.

## Additional Information

**How to cite this article**: Golmakaniyoon, S. *et al*. Cascaded plasmon-plasmon coupling mediated energy transfer across stratified metal-dielectric nanostructures. *Sci. Rep*. **6**, 34086; doi: 10.1038/srep34086 (2016).

## Supplementary Material

Supplementary Information

## Figures and Tables

**Table 1:** Experimental and theoretical steady state efficiency of SP-NRET.

Structure		Stratified/Single layer thickness (nm)	SP-NRET efficiency (%) (Experimental)	SP-NRET efficiency (%) (Theoretical)
Stratified	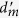 = 20 nm	50	18.15 ± 1.03	24.69
		60	21.74 ± 0.75	27.81
		70	18.64 ± 0.74	25.58
		80	17.27 ± 0.91	23.11
	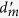 = 30 nm	70	19.06 ± 0.78	25.54
		80	22.38 ± 1.20	27.96
		90	26.46 ± 1.87	29.71
		100	25.59 ± 1.14	24.56
Single layer		40	15.46 ± 0.80	20.07
		60	14.68 ± 1.41	17.66
		90	8.55 ± 0.95	10.56

The stratified layer thickness in stratified structure equals to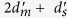; e.g. in the stratified structure with the plasmonic layer thickness of 70 nm and 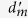 = 20 nm, the spacer layer thickness is 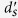 = 30 nm. The plasmonic layer thickness in single layer sample equals to *d*_*m*_. The errors on fitting parameters in steady state efficiency of SP-NRET are estimated to be approximately 8%.

**Table 2:** Time resolved analysis.

Structure	Stratified/Single layer thickness (nm)	t_1_ (ns)	t_2_ (ns)	A_1_	A_2_	t_avg_ (ns)	t_avg_ (ns) (Theoretical)
Stratified	60 ( 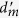 = 20 nm)	0.73 ± 0.05	3.86 ± 0.15	7.98E6	18655	0.85	0.95
	90 ( 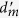 = 30 nm)	0.78 ± 0.11	3.85 ± 0.24	1.35E7	43264	0.89	0.99
Single layer	40	1.52 ± 0.28	6.98 ± 0.48	38522	3085	2.05	2.04
	60	1.48 ± 0.12	6.85 ± 0.51	30514	2986	1.95	2.12
	90	1.41 ± 0.17	6.90 ± 0.36	36368	3310	1.96	2.13

The errors on fitting parameters in time resolved spectroscopy are estimated to be approximately 6%.
