# Supplementary material for: Cascaded plasmon-plasmon coupling mediated energy transfer across stratified metal-dielectric nanostructures
Source: Sci Rep. 2016 Oct 4;6:34086. doi: 10.1038/srep34086 (PMC5048420; doi:10.1038/srep34086)
Supplement: Supplementary Information [file srep34086-s1.pdf]

Cascaded plasmon-plasmon coupling mediated energy transfer across stratified metal-dielectric nanostructures

Sepideh Golmakaniyoon, Pedro Ludwig Hernandez-Martinez, Hilmi Volkan Demir and Xiao Wei Sun

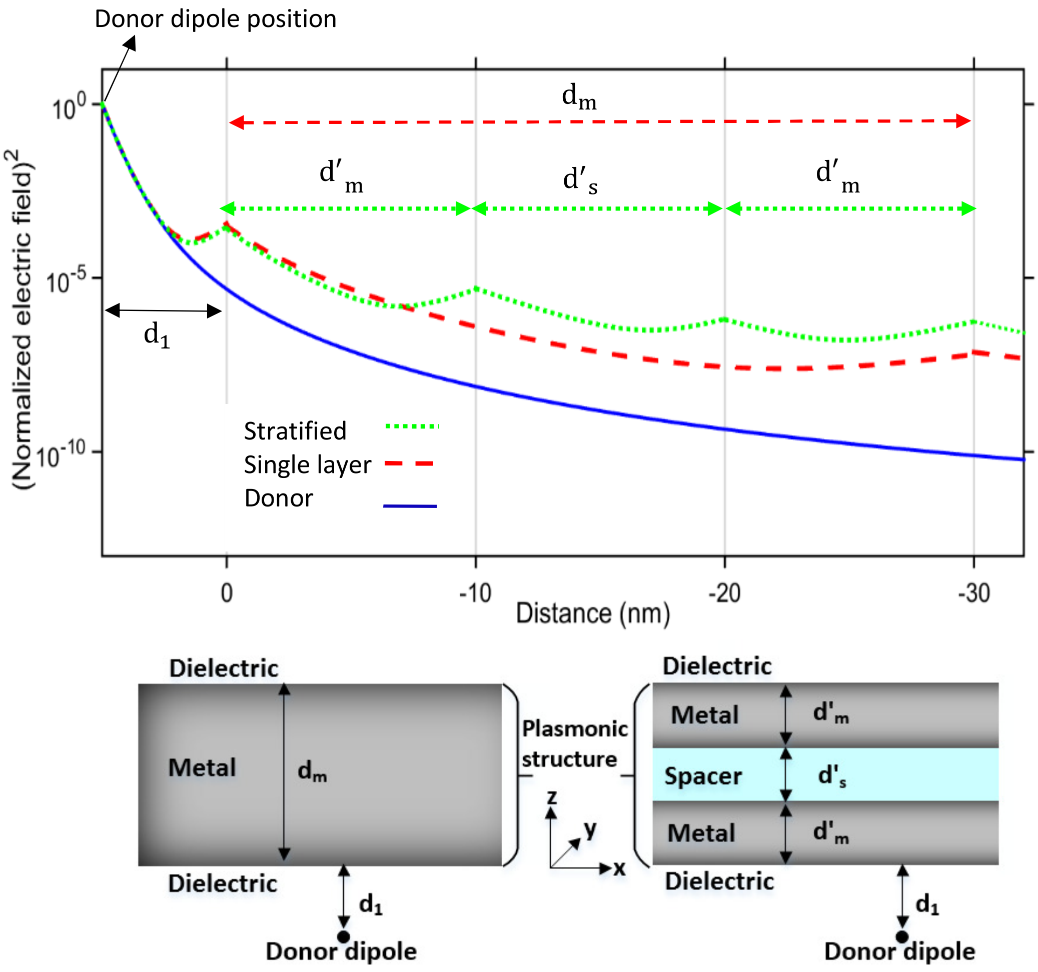

Supplementary Figure 1

Electric filed distribution

Electric field distribution at the plasmonic resonance wavelength of 380 nm through each layer in stratified (green dotted line) and single layer (red dashed line) structures, and the structure without plasmonic layer (blue solid line) for perpendicular dipole with respect to the surface of the metal layer. The donor dipole is placed in front of the plasmonic layer with the distance of d_1.

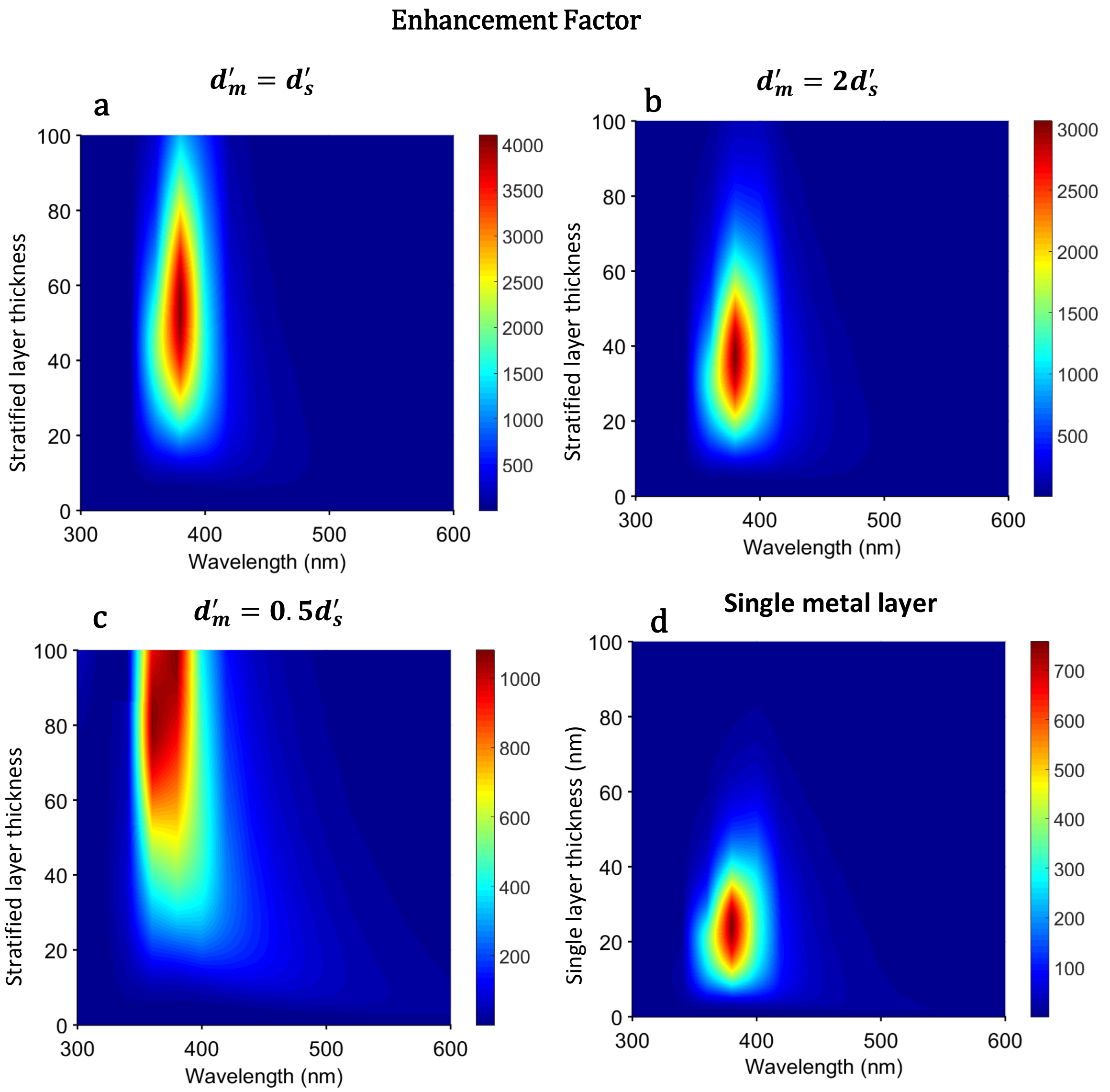

Supplementary Figure 2

Enhancement factor versus spacer layer thickness

The enhancement factor contour at the acceptor position for three stratified plasmonic layers (**a-c**) and the single metal film configuration (**d**), with plasmonic layer/metal film thickness and the excitation wavelength as variables. The plasmonic layer/metal film thickness varies from 0 to 100 nm for stratified and single metal layer structures, and the donor and acceptor dipole distance to the nearest metal film is selected as 5 nm. The donor dipole is perpendicular with respect to the plasmonic layer surface. The thickness of metal nano-films (d'_m) and the spacer layer (d'_s) is as shown: **(a)** $d'_m = d'_s$, **(b)** $d'_m = 2d'_s$ and **(c)** $d'_m = 0.5d'_s$ for the three stratified samples. As can been seen the maximum enhancement factor is obtained in the stratified structure with the distribution of $d'_m = d'_s$. Noted that regardless of the dielectri layer thickness, the enhancement factor is increased in stratified nanostructures compared to the single metal film configuration.

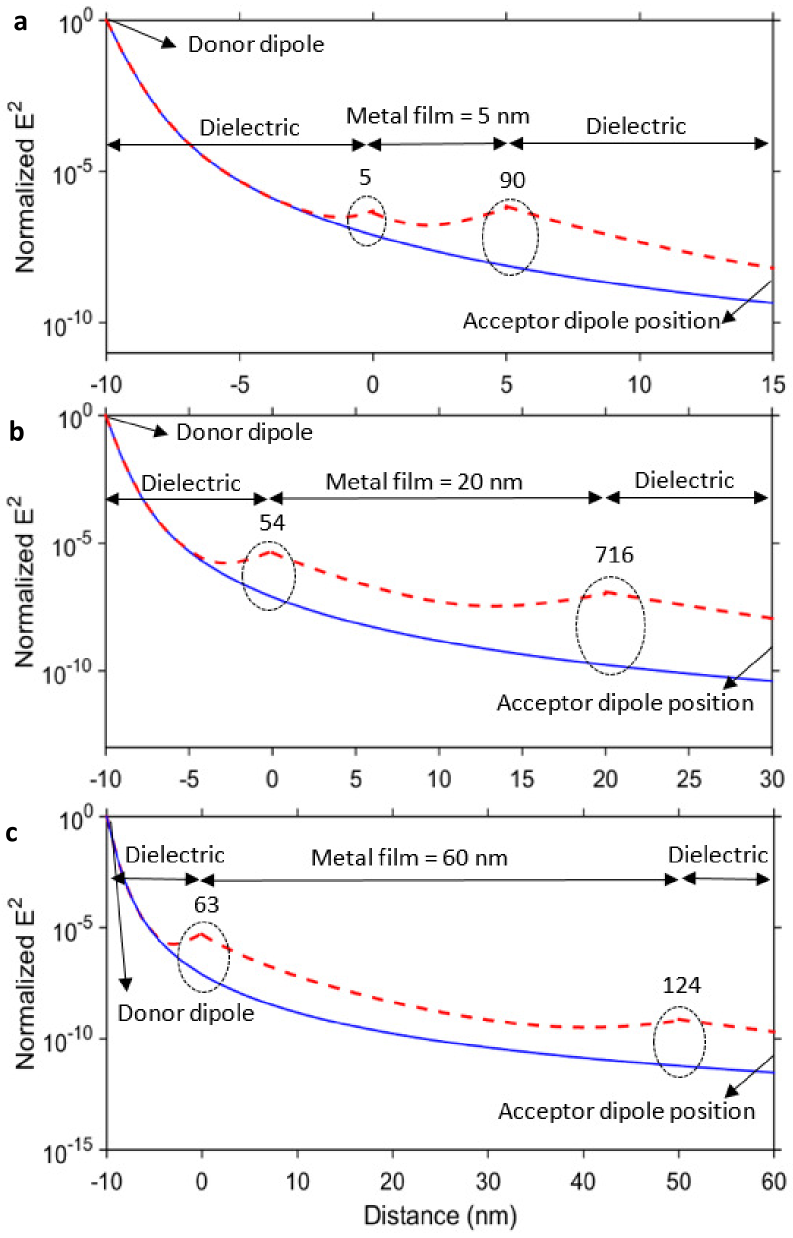

Supplementary Figure 3

Electric field distribution for an optimized metal film thickness

Electric field distribution at the plasmonic resonance wavelength of 380 nm through each layer in the single layer (red dashed line) structures, and the structure without plasmonic layer (blue solid line) for perpendicular dipole with respect to the surface of the metal layer. The donor dipole is placed in front of the plasmonic layer at the distance of 10 nm. The Electric field enhancement with respect to the dipole emission at the metal-dielectric interfaces were shown on the dashed circles for the single layer structure with the silver film thickness of 5 (**a**), 20 (**b**) and 50 (**c**) nm. Donor dipole is placed at the position of -10 nm and the acceptor dipole distance to the nearest metal interface is 10 nm.

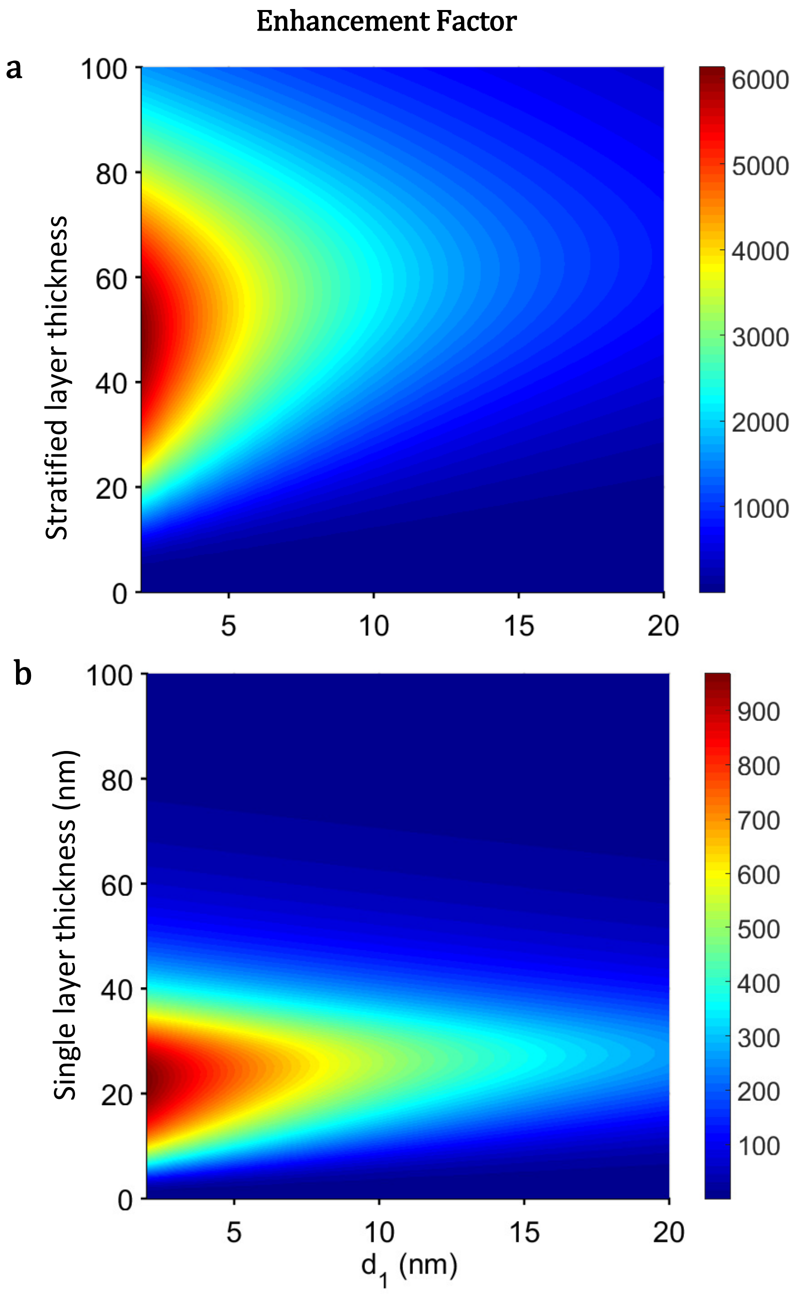

Supplementary Figure 4

Enhancement factor versus donor dipole distance

The enhancement factor contour simulated analytically for the stratified (**a**) and the single layer (**b**) nanostructures versus plasmonic layer/metal film thickness and the donor dipole distance to the nearest metal film surface (d_1), at the acceptor position ($d_2 = 5$ nm). The results demonstrate that the increased enhancement factor in the stratified structure compared to the single layer configuration is independent on d_1. The plasmonic wavelength of 380 nm is chosen for the excitation wavelength and the equal distribution of metal-spacer layers are taken for the stratified nanostructure.

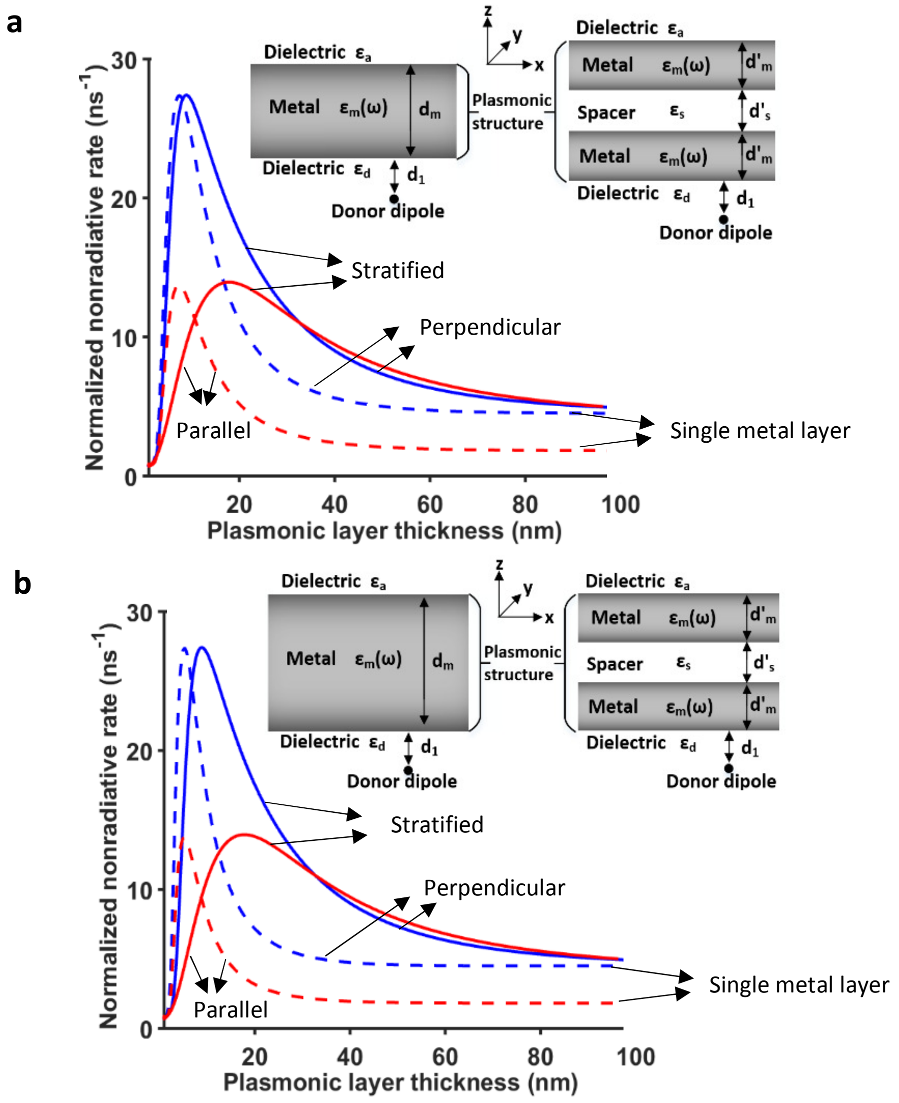

Supplementary Figure 5

Normalized nonradiative rate

The simulation of the normalized nonradiative decay rate (with respect to an intrinsic nonradiative decay rate) for perpendicular (blue line) and parallel (red line) donor dipoles in single layer (dashed line) and stratified (solid line) nanostructures when **(a)** the metal thickness in the single layer structure is equal to the total metal thickness in stratified structure ($d_m = 2\,d'_m$) and **(b)** the metal thickness in the single metal layer structure is equal to the plasmonic layer thickness in stratified structure ($d_m = 2\,d'_m + d'_s$). The plasmonic layer thickness varies from 0 to 100 nm and the donor dipole distance to the nearest metal surface (d_1) is selected as 10 nm. As can be seen, regardless of the comparison method of the plasmonic layer thickness in the single layer and the stratified structure, the donor dipole in stratified plasmonic configuration shows a higher nonradiative decay rate.

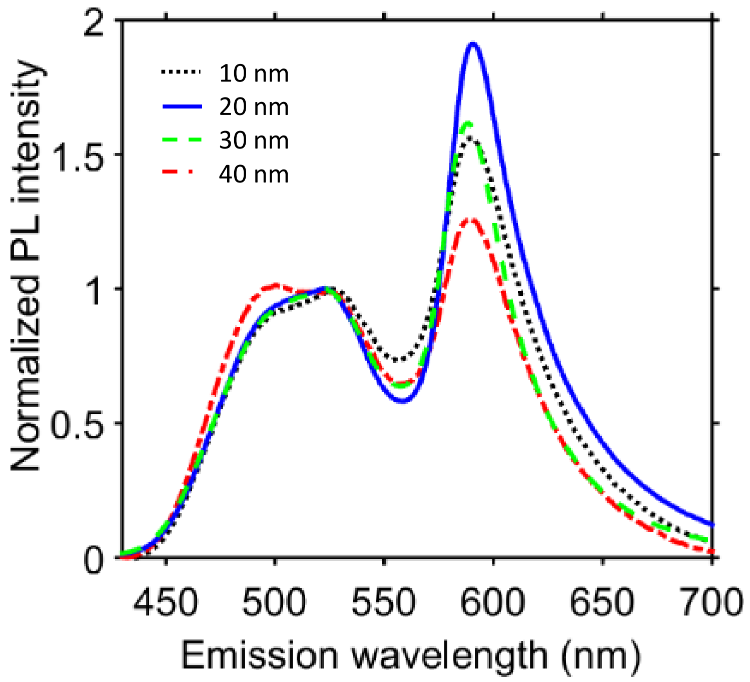

Supplementary Figure 6

SP-NRET acceptor emission versus spacer layer thickness

The normalized PL spectra with respect to the donor emission peak, for the stratified samples with d'_m = 20 nm. The spacer layer thickness (d'_s) varies from 10 to 40 nm. As can be seen that the highest acceptor emission is obtained at a spacer thickness of 20 nm, which is in good agreement with the numerical simulation.

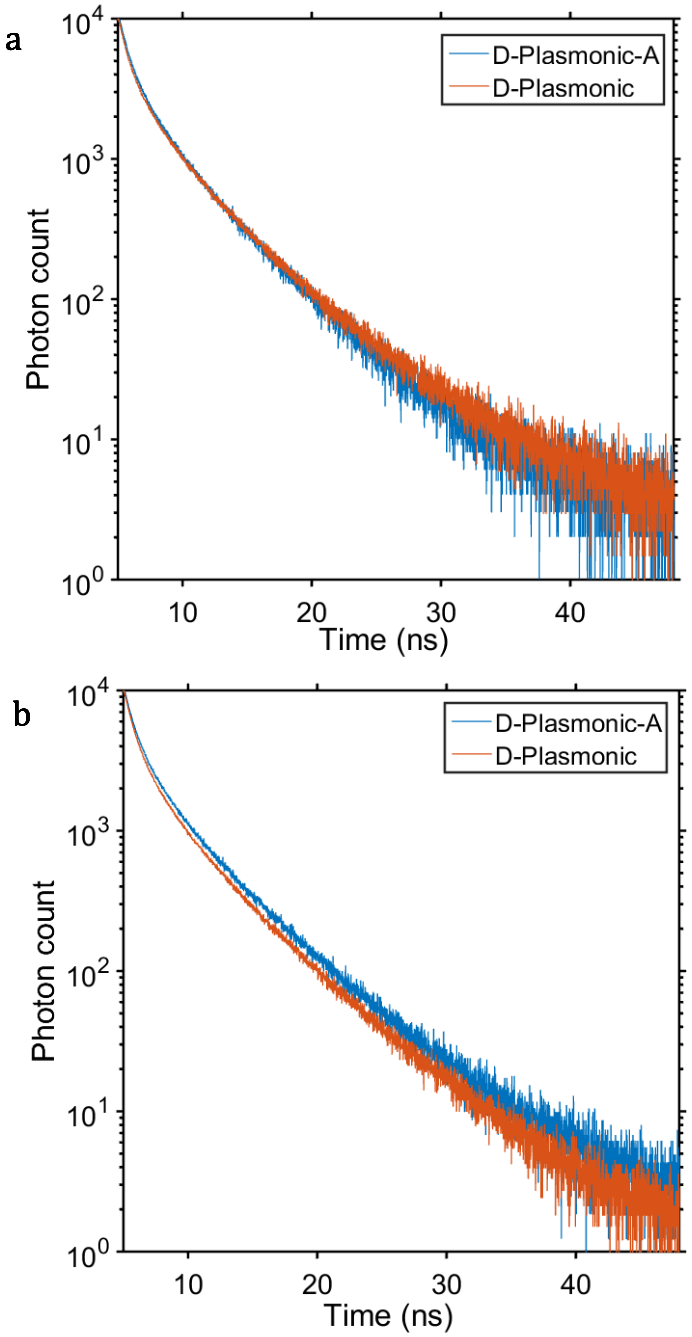

Supplementary Figure 7

Time resolved characterization

Time resolved photoluminescence decay curves with two-exponential decay model at the donor emission wavelength for stratified samples with (D-Plasmonic-A) and without (D-Plasmonic) the acceptor layer, with (**a**) d'_m =20nm and (**b**) d'_m =30nm. In the stratified structures for each configuration, the thicknesses of silver layers and the spacer layer are deposited equally ($d'_m = d'_s$).

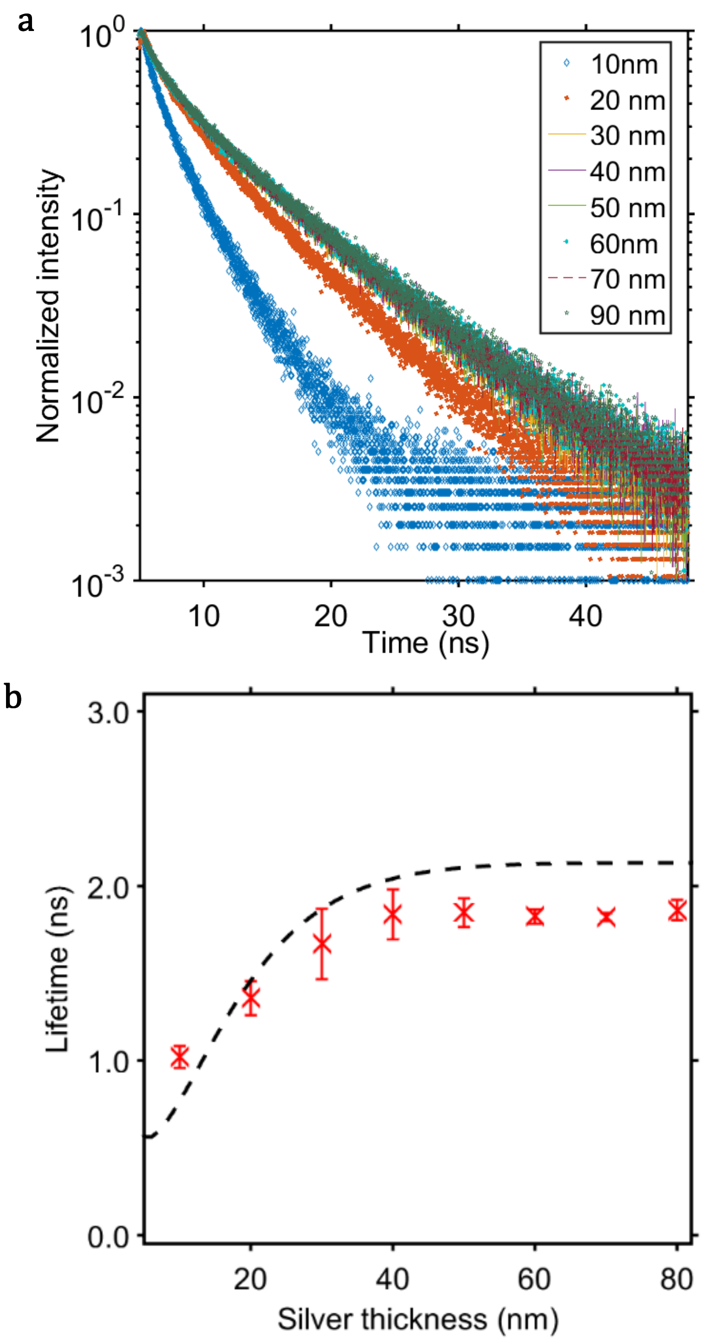

Supplementary Figure 8

Time resolved characterization (experimental and theoretical analysis)

(**a**)Time resolved photoluminescence decay curves with two-exponential decay model at the donor emission wavelength for the single metal layer samples with the silver film thickness of 10 - 90 nm. (**b**) The theoretical calculation of the lifetime (dashed line) and experimental results.

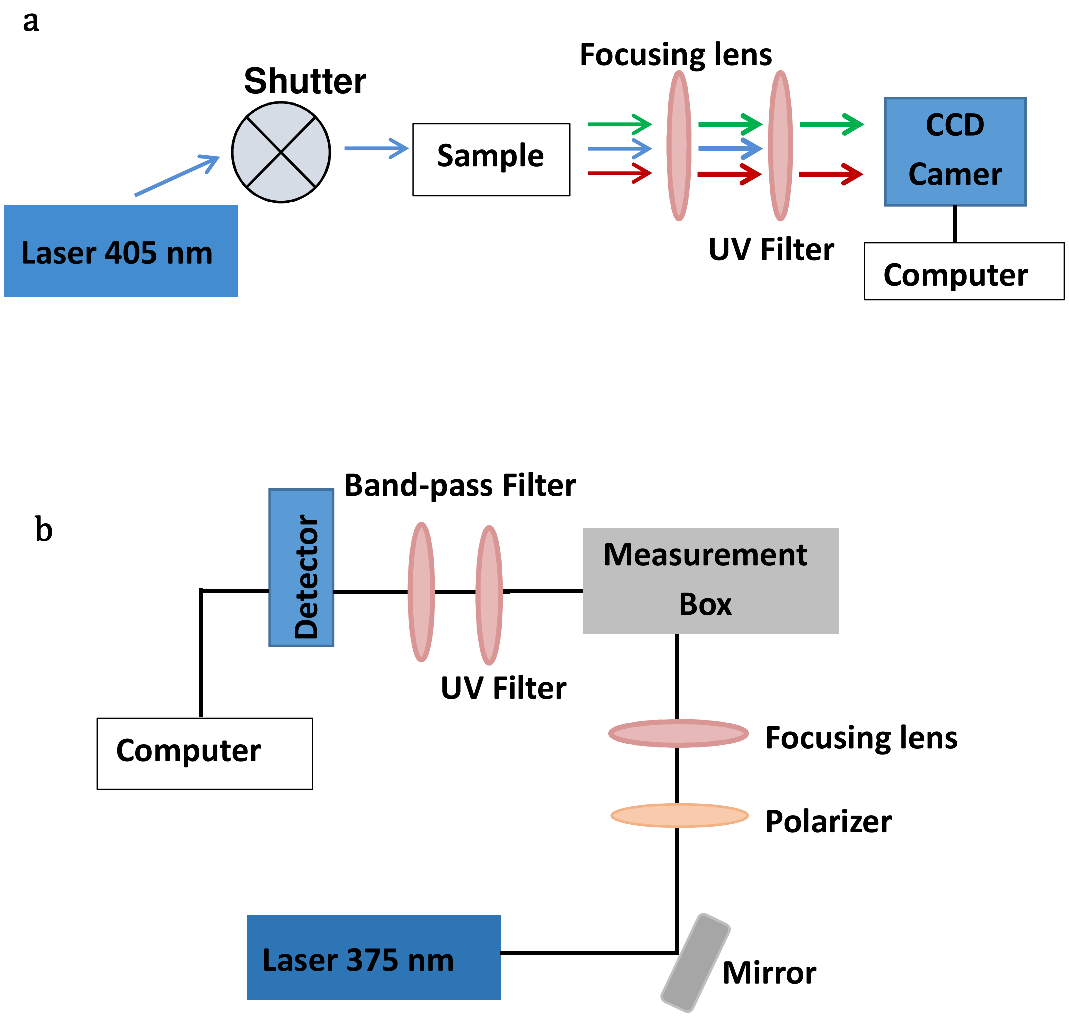

Supplementary Figure 9

Optical setups for photoluminous and time-resolved fluorescence measurements

The schematic illustration of PL intensity measurements setup (**a**) and time-resolved fluorescence system (**b**). In the PL measurement system, the samples are excited by 405 nm laser from a donor side and the resultant intensities are collected from the acceptor side. The UV filter in both systems is used to bypass collection of excitation source from the substrate.

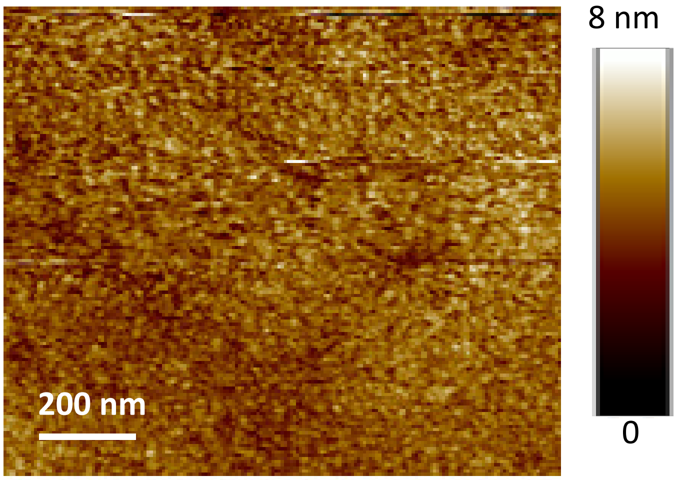

Supplementary Figure 10

Topographic image

Measured surface profiling of silver film in single layer and stratified structures by atomic force microscopy that shows the acceptable flatness.
